# Fear, anxiety, and production in laying hens with healed keel bone fractures

**DOI:** 10.1016/j.psj.2023.102514

**Published:** 2023-01-26

**Authors:** J.L. Edgar, Y. Omi, F. Booth, N. Mackie, G. Richards, J. Tarlton

**Affiliations:** Bristol Veterinary School, University of Bristol, Langford BS40 5DU, United Kingdom

**Keywords:** hen, keel bone fracture, fear, anxiety, welfare

## Abstract

For laying hens, the immediate aftermath and healing period of a keel fracture (**KF**) is characterized by reduced ability to perform species-specific behavior, access resources, and pain. However, the longer-term impacts, once the fracture is completely healed, are less clear. As well as acute pain and behavioral changes, a negative experience can shape future responses to putatively threatening stimuli, raising future fear, and anxiety levels during husbandry-related events. We aimed to determine whether hens that had previously sustained keel bone fractures, but were now outside of the peak age range for new fractures, showed higher fear and anxiety levels compared to intact hens. We also determined if healed keel bone fractures were associated with reduced production, changes in behavior and resource use. One hundred and fifty hens with a palpation score of 1 ( “KF”) and 150 hens with a palpation score of 0 (keel fracture free, “**KFF**”) were selected from a commercial farm at 63 wk of age and housed in 6 groups (3 × KF and 3 × KFF). We compared production (hen weight and feed consumption, egg quantity, quality and weight, floor eggs, shell thickness, and weight) and home pen behavior (behaviors and transitional movements) in both groups. Finally, we measured the responses of KF (*n* = 75) and KFF (*n* = 75) during tonic immobility, novel arena, and novel object tests. KF and KFF hens did not differ in their responses to the tonic immobility, novel arena, and novel object tests, nor were there differences between the 2 groups in home pen behavior and transitional movements. KFF birds were lighter and laid eggs with less eggshell membrane compared KF birds, but no differences were found between KF and KFF in any other production measures. We found no evidence that healed KFs were associated with detrimental welfare effects in laying hens, but further work is required to determine the mechanisms and implications of the lower body weight and egg shell membrane.

## INTRODUCTION

Keel bone damage (**KBD**) is one of the most prevalent problems for commercial laying hens (Riber et al., 2018). The high calcium requirements of continuous egg production in modern layer breeds have led to weakened bones and a high susceptibility to bone fractures (see Riber et al., 2018 for a review), and appear to be especially evident in brown layer genetic lines (Eusemann et al., 2018). The occurrence of keel bone damage is dependent upon both the overall system type and the design of internal housing structures (Wilkins et al., 2011). A recent systematic review of prevalence (at >49 wk of age) across system types suggested a mean fracture prevalence from 23% in conventional cages (range: 0–85%) to 63% in single tier systems (range 33–100%) (Rufener and Makagon, 2020).

The occurrence and immediate aftermath of bone fracture is acutely painful in animals (Minville et al., 2008; Bove et al., 2009; Nasr et al., 2012b). The inflammatory response to fracture can trigger peripheral and central sensitization manifested as hyperalgesia both during and after healing (Bove et al., 2009). In humans, mechanical stimulation of the periosteum (connective tissue covering the bones) is a potent trigger of pain (Santy and Mackintosh, 2001). In hens following a fracture incident, significant soft callus formation is apparent after 2 to 3 wk, with hard callus formation usually completing the healing process after 4 to 6 wk (Richards et al., 2011). Studies have shown effects of keel bone fracture in the early stage of inflammation and repair. These include decreased vertical locomotion (Rentsch et al., 2019) and staying in the nestbox for longer after egg laying (Gebhardt-Henrich and Fröhlich, 2015). However, there has been little work on long-term consequences once the fracture is fully healed.

The most dramatic increase in prevalence of KBD is between the onset and peak of lay (25–35 wk of age) (Harlander-Matauschek et al., 2015), with susceptibility stabilizing after 49 wk of age (Toscano et al., 2018). The majority of studies into the effects of “healed” keel bone damage have focused on this time period (reviewed in Rufener and Makagon, 2020). However, with reports of some fractures (16%) taking several months to heal (Baur et al., 2020), studies of so-called healed fractures may involve birds that are not completely healed, and, because they utilize birds in the peak fracture age range, responses could also be confounded with additional new fractures (which may not necessarily be detectable by palpation; Baur et al., 2020). Whether chronic pain arises following the healing process remains largely unclear but, given the high prevalence of keel bone fracture, this question is of profound relevance to hen welfare and production.

Studies that examined the effects of keel fractures (**KFs**) during the peak susceptibility period for fractures (i.e., those that might be confounded by new fractures) have shown behavioral differences between birds with KFs and control birds. In behavioral tests, 33- to 42-wk-old hens with healed fractures were slower to negotiate a walkway obstacle and made less frequent visits to, and were slower to fly down from, an aerial perch (Nasr et al., 2012a). Other studies comparing the spontaneous behavior of birds with and without KFs had mixed results. Rufener and colleagues (Rufener et al., 2019) investigated use and transitions between 5 zones of a multitier aviary (litter, lower tier, nestboxes, top tier, and wintergarden) in both Lohman brown and Lohman Leghorn birds (21–61 wk old). With increasing keel bone fracture severity, Lohmann brown birds spent more time at the top tier and nestboxes and less time in the litter and lower tier. However, fractures were not linked to the total number of transitions and there was no effect of keel bone status on any aspect of mobility for the Lohmann Leghorns. Rentsch et al. found that, although new fractures decreased vertical locomotion, healed fractures did not affect mobility and neither new nor old fractures were associated with walking pace on ramps (37–39 wk) (Rentsch et al., 2019). Therefore, the effects of truly healed fractures on the birds remain unknown.

Studies involving hens beyond the peak fracture age range are best placed to determine the effect of healed KFs, but there have been mixed results and very few studies. In a longitudinal study throughout the whole laying period, Richards et al. reported a reduction in the use of (raised) popholes among hens that had sustained keel bone fractures, indicating reduced mobility as a result of fracture, especially during colder temperatures (Richards et al., 2012). Another study looked at behavior of 70-wk-old hens (i.e., using birds that are likely to have healed fractures) and found that of all the inactive behaviors, only standing behavior was significantly different between fractured and nonfractured birds (Casey-Trott and Widowski, 2016). Fractured hens spent more time perching and resting on the perches, but the authors attributed this to the fact that hens that use perches are more likely to sustain keel damage. Both studies showed some behavioral differences associated with healed KFs, but it is not clear whether this is attributable to the subjective experience of chronic pain or more simply, a reduction in mobility as a result of physical and physiological changes to the tissue in an around the keel. The avian keel bone provides an anchor to which the muscles for wing motion are attached (Riber et al., 2018) and hard callous formation in the area is likely to reduce control of movement, confounding the use of behavior as an indicator of pain for KF studies.

Drug studies offer the potential to investigate whether healed fractures are currently painful but have produced mixed results and again have often used young birds that may not have been fully healed. For 35-wk-old birds with KFs, the latency to fly down from a perch was reduced by the opioid, butorphanol (Nasr et al., 2012b), but not by the NSAIDs meloxicam and carprofen (Nasr et al., 2015). Using a conditioned place preference paradigm, Nasr showed that 40-wk hens with recently healed fractures preferred an environment that they associated with receiving an opioid analgesic, whereas fracture-free hens did not show the same preference, suggesting the hens with KFs experienced a shift to more positive affective state induced by the opioid, which the authors suggested provides evidence that healed KFs caused pain (Nasr et al., 2013). Rentsch et al. found that 37- to 39-wk fractured hens receiving paracetamol performed less rapid comfort behaviors, but only when the fracture had a visible gap and not when it was healed (Rentsch et al., 2019), indicating that only the “new” fractures were painful.

Although there are some mixed results, the previous studies generally demonstrated effects of KFs which may have welfare implications in terms of a reduced ability to perform species-specific behavior, access resources, and may point to a subjective experience of pain. However, since many of the studies used birds still within the peak fracture window, it is likely that fractures are not fully healed and/or the birds sustained new fractures during the study. Hence the effect of healed fractures on behavior and welfare remains unclear. Additionally, it is not clear whether behavioral changes such as changes in resource use are attributable to the subjective experience of chronic pain or more simply, a reduction in mobility as a result of physical and physiological changes to the tissue in an around the keel.

An additionally highly welfare-relevant but little-studied potential long-term effect of KF is the effect on fear and anxiety. An acutely negative experience such as sustaining a bone fracture has the potential to shape future responses to potentially threatening stimuli, raising general fear, and anxiety levels to husbandry-related events. If this is the case, then sustaining a keel bone fracture will have long-term welfare implications for how birds cope with putatively negative aspects of the commercial environment. Indeed, there is empirical evidence that chronic pain leads to heightened fear and anxiety in animal models. Rats showed increased anxiety-like behaviors including decreased locomotion in an open field test, induced by chronic inflammatory pain (Parent et al., 2012) and a mouse model of neuropathic pain showed anxiety-like behavior in an exploratory (hole board) task (Sieberg et al., 2018). One recent study showed that recent keel bone fractures were associated with an increased duration of tonic immobility and an increased latency to approach a novel object (Wei et al., 2022). Since previous negative experiences in animals act as predictors for future events, and chronic pain is associated with anxiety in other animal models, we hypothesized that hens with healed KFs would be more fearful and anxious than intact hens. Additionally, since most studies measuring changes in behavior, resources use and transitions were from hens within the peak fracture age (Nasr et al., 2012a; Rentsch et al., 2019; Rufener et al., 2019), we also aimed to determine if hens with healed fractures also showed such changes in behavior. We hypothesized that, since hard callous formation on keel bone is likely to reduce control of movement, hens with healed KFs would show a less behaviors requiring use of the muscles anchored to the keel (wingflapping, dustbathing, transitions to perches) compared to keel fracture-free (**KFF**) birds.

## MATERIALS AND METHODS

### Ethics

This project was carried out following ethical approval by the University of Bristol (UIN/18/011) and in accordance with the institutional animal care and use committee (**IACUC**). All hens were rehomed after the study.

### Animals and Housing

Three hundred female Hyline Brown hens were obtained from a commercial-free range farm at 63 wk of age. We selected the age of the study birds to be long past the period of maximum fracture risk (Harlander-Matauschek et al., 2015; Toscano et al., 2018) so the majority of sustained fractures were fully healed and our data were less likely to be confounded by new breaks. At the farm, hens were randomly selected from different locations within the house and were palpated by a trained assessor until 150 hens with a palpation score of 1 (“KF” group) and 150 hens with a palpation score of 0 (“KFF” group) had been selected. Palpation involved collecting the hens, one-by-one in an upright position. To enable the keel area to be exposed for examination, hens were then gently maneuvered to be held by both legs to invert the bird, resting the hen against the researcher's body to reduce pressure on the legs. Palpation involved running 2 fingers down the side of the keel bone, feeling for callus formation indicative of previous damage; with particular attention being paid to the tip of the keel. This was carried out by a trained and validated researcher. Prior training involved practicing on hundreds of dead birds, which were palpated and dissected to validate the accuracy of the overall scoring system (as per Wilkins et al., 2004). Hens with minor fractures which by palpation could not be confidently placed into either category were not included in the study. Hens were transported to Bristol Veterinary School in poultry crates, according to Defra transport regulations. Upon arrival each hen was leg tagged for identification and red mite powder was applied to the feathers before the hen was randomly allocated to their KF or KFF home pen. Three home pens contained KFF birds (50 birds in each) and 3 home pens contained KF birds (50 birds in each).

**Figure 1:**
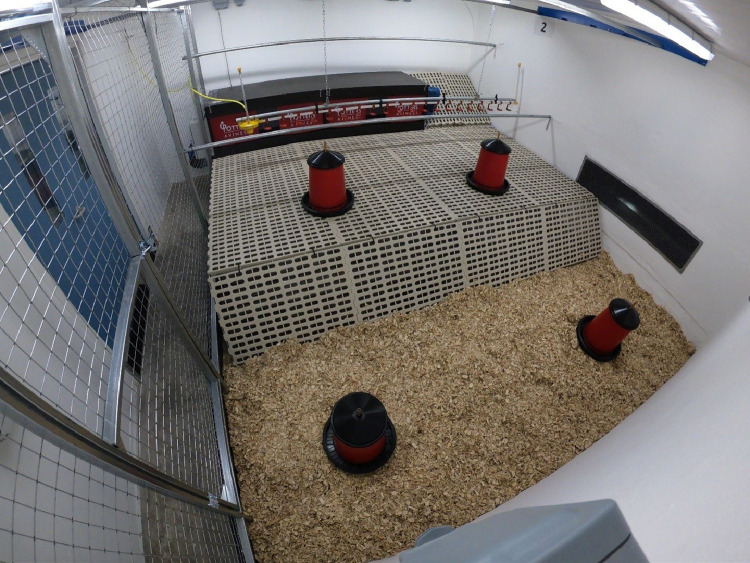
Home pen.

Each of the 6 home pens (4.2 m × 4.4 m × 2.15 m L × W × H) were made up of a litter area and a single tier raised slatted area which was accessible by a slatted ramp (see Figure 1). The raised slatted area contained a nestbox unit (with 4 nestboxes) and 2 metal perches (3.5 cm diameter) at 2 different heights (50 cm and 105 cm above the raised slatted area). Ad libitum layers mash was provided in 4 freestanding poultry feeders; 2 on the raised slatted area and 2 on the litter area. The pens were subject to a 12L:12D lighting schedule.

### Production Measures (Wk 1–4)

The hens were weighed upon arrival and production data were collected on a weekly basis (1 egg collection day per week) as follows:

Egg quality: Eggs were categorized into “first” and “second” eggs by examining their external appearance for 3 traits; calcification, deformity and breaks.

### Floor Eggs: The Number of Eggs Laid Outside the Nestboxes

Egg weight: Ten eggs from each pen were randomly selected and individual weights were recorded once a week (wk 1–4).

Eggshell thickness: From each pen, 5 eggs were randomly selected and labeled. The content of the egg was removed and the remaining eggshell with membrane were washed and left to dry overnight in an incubator (37°C). The following day, the shell thickness was measured using a Vernier caliper with accuracy of 0.001 mm, taken as the mean of measures from 3 points equally distributed around the equator of the eggshell.

Eggshell weights: Eggshells were placed into a furnace set at 700°C for 2 h. This process involved removal of eggshell membrane and other organic material, thereby resulting in accurate eggshell mineral measures. Pre- and postashed eggshell weights were recorded.

Keel bone score: Hens were palpated by a trained assessor so that any KFF hens that sustained a new fracture could be excluded from the individual-level measures. By the end of the study period, 33 out of the 150 intact birds had sustained a new fracture. These were excluded from the individual-level measures, but kept in their pens to maintain the group size and social group.

### Home Pen Behavior Recording (Wk 3)

Home pen behavior was recorded using instantaneous samples at 10 timepoints, 3 times of day: i) morning (09.00–10:00), ii) afternoon (16.00–17:00), and iii) evening (18.20–19:20). Every 5 min, during each of these timepoints a trained observer recorded the number of hens performing behaviors in Table 1, and the number of hens in each location in the pen (see Figure 1).

**Table 1:** Behavioral ethogram (adapted from Nicol et al., 2009).

Behavior	Description
Aggression	Bird directs threat or pecks toward head region of conspecific, sometimes followed by a chase
Body maintenance	Wing flapping, dustbathing, and preening (as above)
Drinking	Pecking and swallowing water from the drinker line
Dust bathing	Performed on the litter area in sitting position, consists of series of behaviors including bill-raking, vertical wing-shaking, litter scratching, and laying on the side and head/body-rubbing
Failed landing	An ascending hen fail to perch, or descending hen colliding with furniture, ground/wall-surface or other hen(s) upon landing
Feeding	Pecking and ingesting layers mash from the feeder
Foraging	Pecking and scratching the ground with beak and legs
Preening	Self-maintaining and manipulation of feathers of the wing and body with the beak. A hen may be sitting, standing, or perching
Peck at enrichment	Pecks at any part of the hanging cabbage
Transition	Movement from one area to another
Standing	Body off the floor with straight legs, upright neck and head
Sitting	Body against the floor with legs tucked underneath (not dustbathing)
Walking/running	Taking steps on the pen floor
Wing flap/Body shake	Wing(s) stretched and flapped multiple times, with/out tail wagging

In addition, continuous observation was used to determine the frequency of the birds’ transitional movements between areas, along with the number of occurrences of rare behaviors (aggression, wing flapping, failed landing), for a 30-min period in the morning, afternoon and evening. A total of 11 transitions recorded were Litter-Slats (**L-S**), Slats-Low perch (**S-LP**), Low perch-Low perch (**LP-LP**), Slats-Nest box (inside) (**S-NBin**), Slats-Nest box (on) (**S-NBon**), Nest box-Low perch (**NB-LP**), Nest box-High perch (**NB-HP**), SlatsHigh Perch (**S-HP**), Low perch-Litter (**LP-Litter**), Low perch-High perch (**LP-HP**), and High perch-Litter (**HP-L**).

### Fear and Anxiety Tests (Wk 4 and 5)

Twenty-five hens per pen (total of 75 KF and 75KFF) were randomly selected to take part in the fear and anxiety tests. The selected hens were used as focal birds for all tests. For all tests, 2 testing rooms of identical size were used (2.9 m × 1.9 m × 2.15 m L × W × H). Testing was conducted between 09:00 and 16:00 with the testing order balanced across the pens.

We used 3 well-validated tests which provided outcome measures in the form of fear and/or anxiety-type responses to 3 different situations (Forkman et al., 2007). The tonic immobility test is a measure of the hens’ innate, antipredator fear response; a response known to be stimulated by, and ameliorated by habituation to, human handling (Jones and Faure, 1981; Bryan Jones and Waddington, 1993; Forkman et al., 2007). The novel arena test provides a measure of the general anxiety of the bird with a strong effect of social isolation/dependence, while the novel object test incorporates neophobic response (Forkman et al., 2007). Hens isolated in a test arena exhibit behaviors thought to be representative of the internal conflict between minimizing detection by predators (e.g., freezing behavior) and the need to regroup with conspecifics (e.g., movement, vocalizations, and escape attempts) (Gallup and Suarez, 1980).

***Tonic Immobility Tests (Wk 4).*** Each hen was collected from their home pen and brought into one of the test rooms. To attempt to induce tonic immobility, the researcher placed the bird into a wooden cradle and gently placed their hands over the head and body of the hen. The hen was restrained in this position for 20 s, after which time the experimenter's hands were slowly moved from the bird. If the bird self-righted within 10 s of release, the induction procedure was repeated, with a maximum of 3 attempts. The number of induction attempts required and the duration of TI (if it occurred) were recorded for each bird. The maximum duration of TI was set at 180 s, at which point the bird was gently removed from the cradle and encouraged to self-right. After TI testing, all birds were keel-palpated for their keel damage status validation at the end of the TI test. If a change in KF status occurred, the current bird was disregarded and a replacement bird was randomly selected from the same pen, and testing commenced with this bird.

***Novel Arena Tests (Wk 5).*** A square novel arena was set up in each test room (1.8 m × 1.8 m × 1.2 m L × W × H). This arena was made from white plastic, with a mesh lid. Each hen was collected from their home pen and immediately placed in the center of the arena. The experimenter withdrew from the room and behavior was recorded for 5 min by an overhead camera. The following variables were assessed from the video recordings: latency to first movement (s), latency to first alarm call(s), number of alarm calls, number of defecations, number of escape attempts. First movement was defined as 2 or more steps in rapid (within 3 s) succession. An escape attempt was defined as the hen attempting to jump and/or fly out of the test arena. Further to the behavioral responses, total distance the hen traveled (cm) and the maximum distance the hen reached from the start point (cm) were calculated by computer software, based on kernelized correlation filters (Henriques et al., 2015).

***Novel Object Test (Wk 5).*** Immediately after the novel arena test, the experimenter entered the test arena, moved the bird to the side of the arena nearest the entrance, and placed a novel object in the center of the arena before leaving the room. The novel object was an inflated plastic turtle toy (24 cm L). For a period of 5 min, the following behavioral responses were assessed from video recordings: duration of freezing (both with and without head movement) (s), latency to first alarm call (s), number of alarm calls, number of defecations, number of pecks at the object, number of escape attempts. Total distance traveled (cm) was also analyzed as for the novel arena test.

### Statistical Analyses

All data were tested for normality and normality of residuals, and a logarithm or arcsine square root transformation was applied if required. For all tests with multiple measures, Bonferroni correction was applied (see Table 2, Table 3, Table 4 for required level of significance). All analysis was performed using IBM SPSS 24.

**Table 2:** Responses of hens to the fear and anxiety tests according to keel fracture status, and effect of keel fracture and pen (required *P* values: tonic immobility *P* = 0.025, novel area *P* = 0.016, novel object *P* = 0.007).

		Keel racture		Keel fracture-free		Effect of keel fracture		Effect of pen		
Measure		Mean	SE	Mean	SE	*F*	*P*	*F*	*P*	df
Tonic immobility	No of attempts	2.22	0.09	2.22	0.92	0.03	0.96	5.77	0.15	1, 151
	Mean duration (s)	64.52	7.49	75.70	7.76	3.14	0.22	9.09	0.10	1, 151
Novel arena	First movement (s)	178.57	13.00	196.94	12.90	0.09	0.77	0.89	0.49	1, 144
	First call (s)	214.32	14.80	187.83	14.87	2.86	0.09	0.88	0.50	1, 139
	No of calls	1.25	0.27	1.76	0.28	2.19	0.14	1.09	0.37	1, 144
	Total distance (cm)	894.94	110.57	817.97	109.30	0.63	0.43	1.72	0.13	1, 144
Novel object	Time spent freezing (s)	37.26	5.00	38.13	4.83	0.33	0.57	2.07	0.07	1, 137
	First call (s)	190.56	15.87	202.24	15.13	1.79	0.18	0.79	0.56	1, 135
	No of calls	5.46	4.21	1.39	0.25	0.06	0.91	0.57	0.72	1, 137
	No of object pecks	0.30	0.13	0.04	0.04	3.29	0.07	1.06	0.39	1, 137
	No of defecations	0.27	0.05	0.21	0.06	2.12	0.15	0.84	0.53	1, 136
	No of escape attempts	0.03	0.02	0.01	0.01	1.06	0.31	1.04	0.40	1, 136
	Total distance (cm)	1410.75	157.32	1290.00	152.52	0.01	0.93	1.98	0.09	1, 137

**Table 3:** Mean (±SE) percentage of hens recorded in locations and performing behaviors according to keel fracture status, and effect of keel fracture, time of day, and interaction effects (required *P* values: location *P* = 0.007, behavior *P* = 0.006, rare behaviors *P* = 0.008).

		Keel fracture		Keel fracture-free		Effect of keel fracture		Effect of time of day		Keel fracture × time of day		
Measure		Mean	SE	Mean	SE	*F*	*P*	*F*	*P*	*F*	*P*	df
Location (% of hens)	Litter	38.58	1.53	45.58	1.49	4.98	0.09	0.92	0.44	0.94	0.43	1, 4
	Slats	39.86	1.50	36.34	1.50	3.23	0.15	6.82	0.02	0.70	0.53	1, 4
	Low perch	9.54	1.59	7.57	1.42	0.40	0.56	41.29	<0.001⁎	0.80	0.48	1, 4
	High perch	1.36	0.59	1.66	0.59	0.27	0.63	11.88	0.004⁎	0.25	0.78	1, 4
	On drinker line	1.31	0.38	0.73	0.25	0.51	0.52	0.20	0.82	0.16	0.86	1, 4
	In nestbox	7.03	1.40	6.62	1.07	0.02	0.90	16.97	0.001⁎	0.88	0.45	1, 4
	On nestbox	2.30	0.40	1.49	0.39	1.20	0.34	7.54	0.01	0.90	0.44	1, 4
Behavior (% of hens)	Body maintenance	6.72	0.96	9.24	2.31	11.98	0.03	10.00	0.01	4.54	0.05	1, 4
	Feed	28.69	1.72	29.19	2.18	0.09	0.78	10.99	0.005⁎	0.35	0.71	1, 4
	Drink	5.74	0.56	5.38	0.64	0.18	0.69	2.92	0.11	0.26	0.78	1, 4
	Forage	10.32	2.30	11.28	2.55	0.35	0.59	33.62	<0.001⁎	0.87	0.45	1, 4
	Sit	6.32	1.05	7.21	1.56	1.26	0.33	12.75	0.003⁎	1.06	0.39	1, 4
	Stand	21.53	1.91	17.28	1.04	3.35	0.14	7.20	0.02	0.59	0.58	1, 4
	Walk/run	8.56	0.80	7.91	0.83	2.28	0.21	24.41	<0.001⁎	0.03	0.97	1, 4
Rare behaviors (number)	Aggression	3.67	1.38	3.11	0.70	0.095	0.77	3.08	0.10	0.86	0.46	1, 4
	Failed landing	0.67	0.24	0.56	0.24	0.1	0.77	1.88	0.21	0.47	0.64	1, 4
	Wing flap	16.67	1.88	13.89	0.89	3.05	0.16	5.71	0.03	2.11	0.18	1, 4

⁎ Indicate statistically significant effects.

**Table 4:** Mean (±SE) number of transitions according to keel fracture status, as well as the effect of keel fracture, time of day, and interaction effects (required *P* value = 0.004).

		Keel fracture		Keel fracture-free		Effect of keel fracture		Effect of time of day		Keel fracture × time of day		
Measure		Mean	SE	Mean	SE	*F*	*P*	*F*	*P*	*F*	*P*	df
Number of transitions	Litter-Slats	115.00	13.25	125.33	15.60	0.88	0.40	227.90	<0.001⁎	2.36	0.16	1, 4
	Slats-Low perch	38.44	8.43	29.22	6.47	2.92	0.16	43.79	<0.001⁎	1.12	0.37	1, 4
	Low perch-Low perch	16.33	3.32	14.78	3.86	0.04	0.84	15.97	0.002⁎	0.83	0.47	1, 4
	Slats-Nest box (inside)	2.11	0.81	1.44	0.78	0.23	0.66	2.40	0.20	2.40	0.20	1, 4
	Slats-Nest box	3.44	1.19	3.67	0.76	0.02	0.90	1.83	0.22	0.83	0.43	1, 4
	Nest box-Low perch	21.44	4.50	14.67	4.31	1.41	0.30	16.92	0.001⁎	0.06	0.95	1, 4
	Nest box-High perch	10.78	2.71	8.33	3.46	0.45	0.54	12.10	0.004⁎	0.84	0.47	1, 4
	Slats-High Perch	0.22	0.15	0.22	0.22	2.00	0.23	1.40	0.30	0.60	0.57	1, 4
	Low perch-Litter	0.22	0.22	0.111	0.111	0.20	0.68	0.60	0.57	1.40	0.30	1, 4
	Low perch-High perch	0.00	0.00	0.44	0.24	16	0.016	4.00	0.02	4.00	0.02	1, 4
	High perch-Litter	0.22	0.22	0.11	0.11	0.20	0.68	3.00	0.54	3.00	0.54	1, 4
	Total transitions	208.22	29.91	198.33	27.27	0.19	0.69	181.05	<0.001⁎	1.45	0.29	1, 4

⁎ Indicate statistically significant effects.

***Fear and Anxiety Tests.*** For TI data, a univariate general linear model (**GLM**) was used to compare duration of TI and the number of induction attempts needed between treatment groups (KF and KFF), with keel fracture status (**KFS**) as a fixed factor and pen as a random effect. For NA and NO tests, frequencies of behaviors (number of alarm calls, escape attempts, defecation, object pecking (for NO)) were compared between KF and KFF using a univariate GLM, with KFS as a fixed factor and pen as a random effect. Similarly, the effect of both KFS and pen was determined on latency to first move, latency to first vocalization, duration of freezing, total distance traveled during NA and NO tests and maximum distance traveled during NA test.

***Home-Pen Behaviors and Hen Location.*** Frequency counts of the number of hens performing each behavior and at each location within the ethogram were converted into the mean (of the 10 scans) percentage of hens performing each behavior and at each location. The number of transitions and rare behaviors were recorded for each pen at each time of day. A repeated measures GLM was performed, with time of day (am, pm, and evening) as a within subjects factor and treatment (KF and KFF) as a between subjects factor. Pen was included as a random effect.

***Productivity Measures.*** Hen weight, feed consumption per hen/day, egg shell thickness, egg shell weight, and percentage of egg shell membrane of KF and KFF hens were compared using a One-way GLM with treatment (KF and KFF) used as a fixed effect and pen as a random factor. The number of eggs per hen/day, percentage of first quality eggs, percentage of floor eggs and egg weight of KF and KFF were compared using a repeated measures GLM with date as a within subjects factor and treatment (KF and KFF) as a between subjects factor.

## RESULTS

### Fear and Anxiety Tests

There was no effect of KFS or pen on the hens’ responses during the tonic immobility test, novel arena test, and novel object test (see Table 2 for means and other statistics).

### Home Pen Behavior

Statistics relating to behavior and location of the birds is shown in Table 3. There was no effect of KFS on behavior, location, and transitions, and no interaction effect between KFS and time of day on these measures.

**Figure 2:**
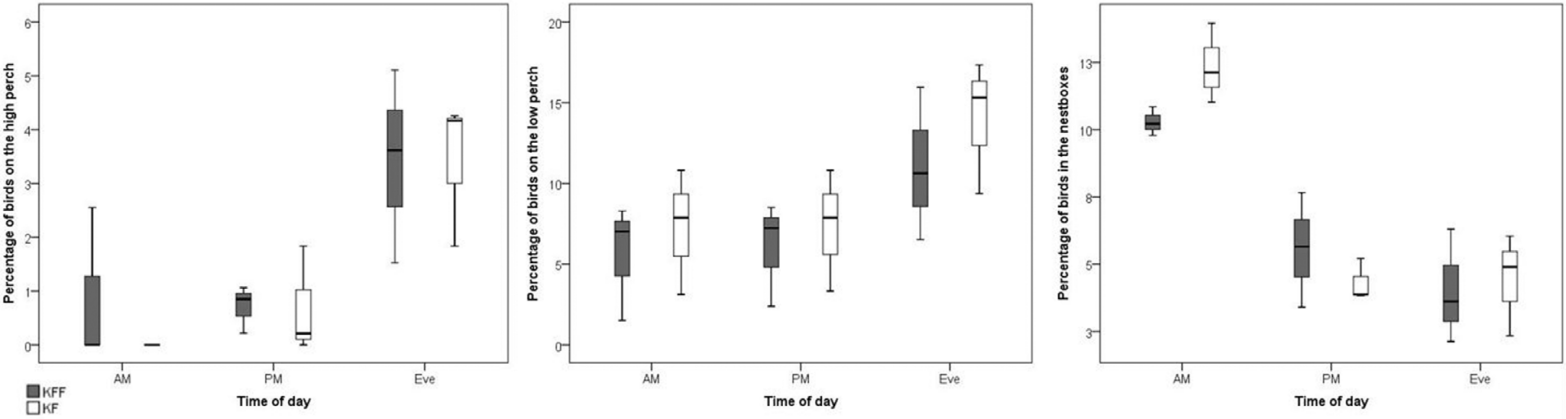
Percentage of birds at locations according to keel fracture status and time of day.

However, there was an effect of time of day on use of the high perch (*F* = 11.88, *P* = 0.004), low perch (*F* = 41.29, *P* < 0.001), and in the nestbox (*F* = 16.97, *P* = 0.001) (see Figure 2). Specifically, more birds were observed on the high perch in the evening when compared to the morning (*P* = 0.016). More birds were observed on the low perch in the evening when compared to the morning (*P* = 0.001) and afternoon (*P* = 0.004). More birds were observed inside the nestbox in the morning when compared to the afternoon (*P* < 0.001) and evening (*P* = 0.001).

**Figure 3:**
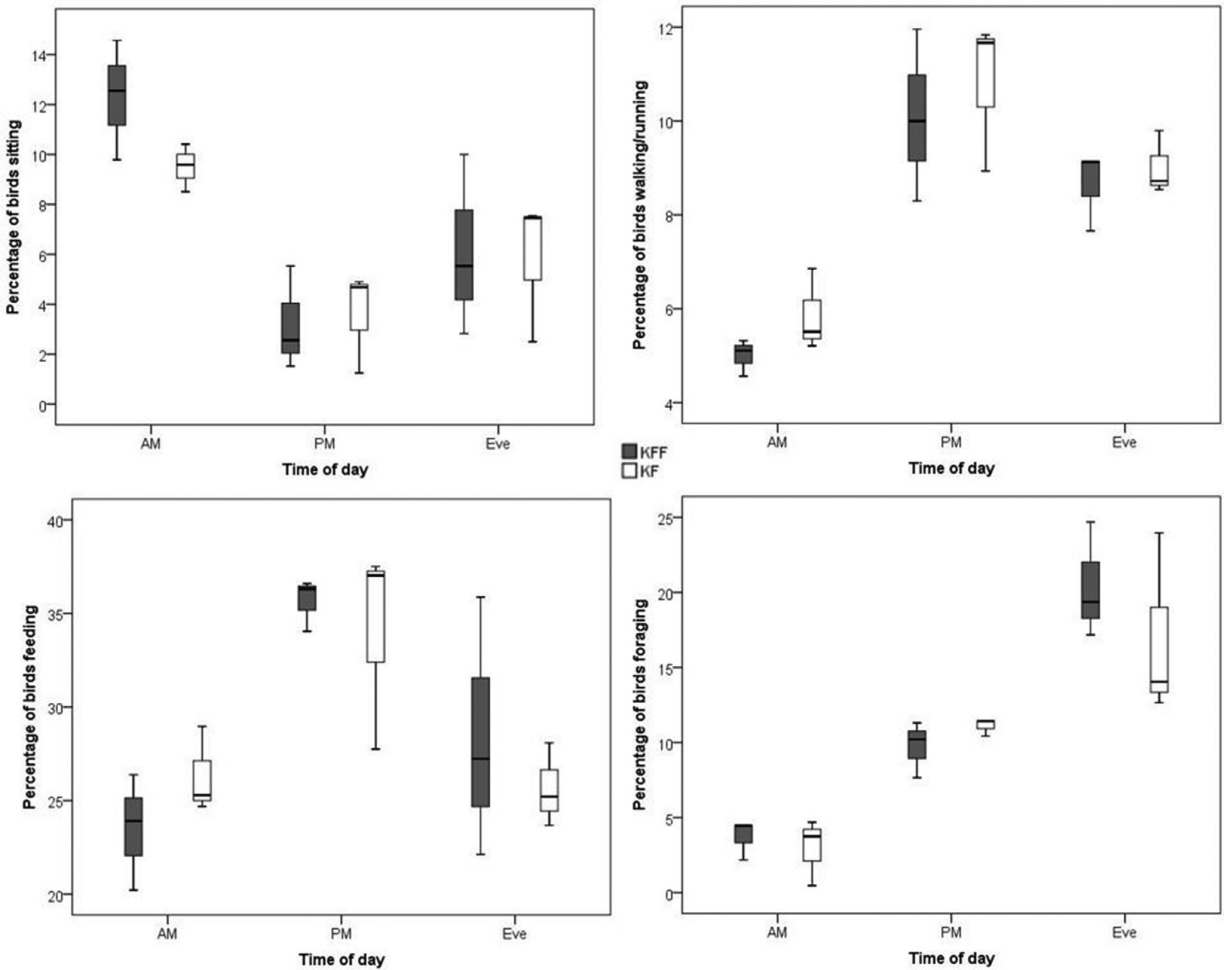
Percentage of birds performing behaviors according to keel fracture status and time of day.

There was also an effect of time of day on sitting (*F* = 12.75, *P* = 0.003), walking/running (*F* = 24.41, *P* < 0.001), feeding (*F* = 10.99, *P* = 0.005), and foraging (*F* = 33.62, *P* < 0.001) (see Figure 3).

There was an effect of time of day on the total number of transitions between the litter and slats (*F* = 227.90, *P* < 0.001), slats and low perch (*F* = 43.79, *P* < 0.001), low perch and (other) low perch (*F* = 15.97, *P* = .002), nest box and low perch (*F* = 16.92, *P* = 0.001), nestbox and high perch (*F* = 12.10, *P* = 0.004) as well as the total number of transitions (*F* = 181.05, *P* < 0.001) (see Table 4).

### Production

**Figure 4:**
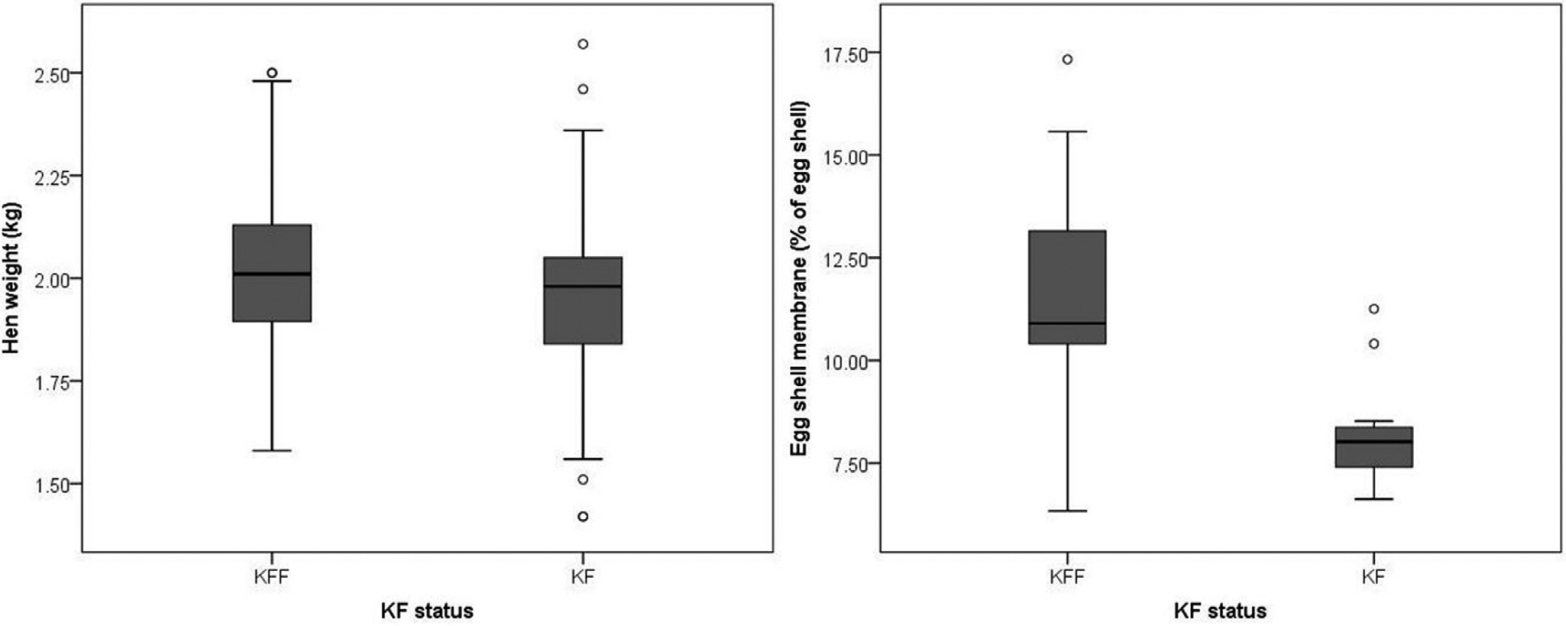
Hen weight and egg shell membrane according to keel fracture status.

Table 5 shows the statistics relating to production. There was an effect of KFS on hen weight, with KFF hens being heavier than KF hens (*F* = 5.94, *P* = 0.015). There was an effect of KFS on egg shell membrane, with KFF birds laying eggs with higher egg shell membrane weight than KF birds (*F* = 31.51, *P* < 0.001) (see Figure 4).

**Table 5:** Production data according to keel fracture status, and effect of date and interaction effects (if appropriate) (required *P* values; hen weight *P* = 0.05, feed consumption *P* = 0.05, egg production *P* = 0.008).

		Keel fracture		Keel fracture-free		Effect of keel fracture		df	Effect of date		df	Interaction effect		
Measure		Mean	SE	Mean	SE	*F*	*P*		*F*	*P*		*F*	*P*	df
Hen weight (kg)		1.94	0.02	2.01	0.01	5.94	0.015⁎	1, 289						
Feed consumption/hen/week (g)		0.15	0.00	0.15	0.00	0.01	0.92	1, 4	2.00	0.031⁎	1, 3	2.00	0.19	1, 3
Egg production	Proportion of 1st quality	0.92	0.03	0.88	0.02	1.15	0.34	1, 4	0.02	0.88	1, 3	1.50	0.29	1, 3
	Proportion of floor eggs	0.02	0.01	0.01	0.01	0.49	0.52	1, 4	1.83	0.25	1, 3	0.27	0.63	1, 3
	Egg weight (g)	66.46	0.75	65.43	0.76	2.49	0.12	1, 58	2.00	0.12	1, 3	0.46	0.71	1, 3
	No of eggs per hen/day	0.91	0.01	0.89	0.02	0.80	0.42	1, 4	22.15	<0.001⁎	1, 19	0.35	0.99	1, 19
	Egg shell thickness (mm)	0.40	0.00	0.40	0.01	0.00	0.98	1, 54						
	Egg shell weight (g)	6.10	0.05	6.20	0.03	0.46	0.50	1, 54						
	Egg shell membrane (%)	8.15	0.53	11.79	1.16	31.51	<0.001	1, 54						

⁎ Indicate statistically significant effects.

There was no effect of keel bone fracture status on any other measured egg quality data, including egg shell thickness and egg shell weight, and effect of KFS nor date on the on proportion of first quality eggs, floor eggs, egg weight, number of eggs per hen/day, and feed consumption per hen/week.

## DISCUSSION

We aimed to determine whether hens with healed keel bone fractures showed higher levels of fear and anxiety compared to hens with intact keels. We hypothesized that hens with a healed keel bone fracture would be more fearful and anxious than intact hens. Contrary to this hypothesis, we found that hens with healed KFs did not differ to intact hens in their responses during the tonic immobility, novel arena, and novel object tests. All 3 fear tests measure responses to commercially relevant stimuli, since on farms the birds are likely to encounter novel stimuli and situations (e.g., changing environments, new people entering the house, potential predators). The lack of effect of KFS on the birds’ responses during these tests indicates that, after healing, KFs are not associated with chronic pain that manifests in increased fear and/or anxiety within these contexts, nor has their experience of sustaining a keel bone fracture resulted in heightened fear or anxiety to these stimuli. This finding is in keeping with Armstrong et al., who recently found that downregulation of adult hippocampal neurogenesis—a biomarker of chronic stress—occurred following a KF, but crucially, this only persisted for 3 to 4 wk after the fracture event (Armstrong et al., 2020).

In determining the relationship between KFS and spontaneous bird behavior, we also found no effect of KFS on transitions between different areas of the pen. This finding is in keeping some studies that showed no link between healed fractures and general transitions between areas (Rentsch et al., 2019), although in contrast to others that showed some reduced vertical movements (Nasr et al., 2012a; Richards et al., 2012). It has been hypothesized that keel bone fracture reduces the hens ability to move around their environment due to reduced mobility and/or pain. Our home pens were set up so that birds needed to traverse (up and down) the ramp to access food, water, and nestboxes but that it was less essential that they traversed between the low and high perches (because there were no essential resources there). If the healed KF were causing pain and/or reduced mobility we would expect to see no differences between KF and KFF in transitions up the ramp (to access essential resources) but differences between the groups in transitions between the high and low perches, or on the top of the nestboxes (where there are no essential resources). Since we found no differences between KF and KFF groups in both types of transitions, we can conclude that the healed KFs did not cause pain and reduced mobility that manifested in ability to access essential and nonessential resources.

We found no other effects of healed KFS on behaviors or use of the resources between our groups. This included body maintenance (preening and dustbathing); an activity that requires vigorous movement of the body, which might be restricted due to reduced mobility and/or pain. In addition, one would expect walking and foraging (in the litter) to be affected by keel bone status, but we found no such association. However, there remains the possibility that our study design failed to pick up keel bone effects on behavior.

In terms of production measures, we found that hens with healed KFs were lighter than the control hens. This could be caused by a residual effect from increased energy demands caused by the immediate aftermath of energy and nutrients being redirected toward the fracture, or a residual effect from behavioral changes immediately following the fracture event. However, our study design, whereby we used birds that had already sustained fractures means that we cannot infer causality and lighter birds might have been more likely to break their keels in the first place due to less tissue protecting the keel or because of some behavioral features that caused both characteristics. This raises the question as to whether we should have selected control birds that were matched for weight with a matched KF bird, but this selection would also have led to bias in our study sample, by only selecting birds at the heavier end of the KF mean weight.

There was no effect of KFS on the measures of egg quality, including the number of first and second quality eggs, number of floor eggs, egg weight, and shell thickness. Nasr et al. found that hens with healed KFs laid eggs with lower eggshell weight compared to intact hens, and that KF severity had a negative relationship with egg weight and surface area (Nasr et al., 2012a). It would be hypothesized that tissue damage would redirect energy and resources, particularly calcium, away from egg development. In fact, we did find a significant effect of KFS on egg shell weight lost in the ashing process. The ashing process removes organic matter from the egg shell; much of which is in the egg shell membranes. The 2 egg shell membranes are essential for the formation of eggshell and then act to retain the albumen and prevent penetration of bacteria (Nakano et al., 2003). The organic matter of eggshell and shell membranes contains proteins as major constituents with small amounts of carbohydrates and lipids (Burley and Vadehra, 1989). The finding that the birds with a healed KF had reduced egg shell membrane compared to intact birds could be caused by the residual effects of increased metabolic requirements of the damaged bone. During the healing process, energy, nutrients, and minerals that are usually used in egg production are redirected to the bone healing process (Thiruvenkadan et al., 2010), although an effect on the egg shell membrane has, to our knowledge, not been investigated or reported before. Since the egg shell membrane plays an important role in protecting the egg contents, further research is required to determine the prevalence and the effects this has on egg and chick quality. If KFs chronically reduce egg shell membrane quantity and quality then there may be implications in layer breeder farms in the form of reduced hatchability and quality of chicks.

Our use of palpation and categorization of birds into binary categories (which facilitated housing birds together in multiple groups of the same category) meant that we were not able to take into account the complexities of the KF such as precise location, severity and type. These factors can be determined using radiography (Rentsch et al., 2019) but were not deemed practical in the current study due to the need for collecting large numbers of birds from a commercial farm. Instead, we opted for palpation by assessors who had been trained using validation with dissection (as per Wilkins et al., 2004). In addition, we have added further info on the hens with minor fractures which by palpation could not be confidently placed into either category were not included in the study. Additionally, the nature of our study, which involved collecting birds after KFs had occurred and had healed, means that we are not able to infer causality; an issue also raised by previous authors (e.g., Riber et al., 2018). Individual differences in behavior or fear may already be evident before fracture. For example, birds that are more fearful, may be more likely to suffer a KF. For the current study, all birds in a room were either fracture score 1 or 0—this allowed the observation of group-level behavior but the grouping of birds all the same fracture status may result in different social and behavioral dynamics compared to on a commercial farm. As was expected, during the study period, some of the intact birds sustained keel bone fractures (33 out of the 150 intact birds by the end of the study period). As our primary aim was to determine the relationship between KFS and fear/anxiety, these birds were excluded from the individual-level fear tests, although they remained as part of the social group for the group-level data, since we decided it was important to retain a stable social group. To determine the association between KF and spontaneous behavior, a more robust future study might involve individual monitoring of the birds to take measures before and after keel bone fracture. However, individual monitoring of the large numbers of birds required to ensure sufficient birds transition from KFF to KF would be difficult and costly. The design of our study meant that we were able to monitor group-level productivity and behavior as well as individual level fear levels in birds with mostly old fractures. Rentsch et al. investigated behavior before and after KF and found no differences in activity (vertical locomotion, walking pace, rapid comfort behavior, and feather maintenance combined) between birds that later sustained a fracture and those that didn't (Rentsch et al., 2019), suggesting the fracture event is causal for the behavior rather than the other way around. Since we found no difference in fear or anxiety responses between the 2 groups, we can conclude that healed fractures are unlikely to have had an effect on long-term fear and anxiety levels in our birds.

## CONCLUSIONS

Previous literature has demonstrated that KFs are associated with pain and behavioral changes persisting for weeks after the fracture. We found no evidence that, once healed, KFs were associated with detrimental welfare in laying hens. However, further work is required to determine the mechanisms and implications of the reduced body weight and egg shell membrane.

## References

[bib0001] Armstrong E.A., Rufener C., Toscano M.J., Eastham J.E., Guy J.H., Sandilands V., Boswell T., Smulders T.V. (2020). Keel bone fractures induce a depressive-like state in laying hens. Sci. Rep..

[bib0002] Baur S., Rufener C., Toscano M.J., Geissbühler U. (2020). Radiographic evaluation of keel bone damage in laying hens—morphologic and temporal observations in a longitudinal study. Front. Vet. Sci..

[bib0003] Bove S.E., Flatters S.J.L., Inglis J.J., Mantyh P.W. (2009). New advances in musculoskeletal pain. Brain Res. Rev..

[bib0004] Bryan Jones R., Waddington D. (1993). Attenuation of the domestic chick's fear of human beings via regular handling: in search of a sensitive period. Appl. Anim. Behav. Sci..

[bib0005] Burley R.W., Vadehra D.V. (1989). The Avian Egg: Chemistry and Biology.

[bib0006] Casey-Trott T.M., Widowski T.M. (2016). Behavioral differences of laying hens with fractured keel bones within furnished cages. Front. Vet. Sci..

[bib0007] Eusemann B.K., Baulain U., Schrader L., Thöne-Reineke C., Patt A., Petow S. (2018). Radiographic examination of keel bone damage in living laying hens of different strains kept in two housing systems. PLoS One.

[bib0008] Forkman B., Boissy A., Meunier-Salaun M.C., Canali E., Jones R.B. (2007). A critical review of fear tests used on cattle, pigs, sheep, poultry and horses. Physiol. Behav..

[bib0009] Gallup G.G., Suarez S.D. (1980). An ethological analysis of open-field behaviour in chickens. Anim. Behav..

[bib0010] Gebhardt-Henrich S.G., Fröhlich E.K. (2015). Early onset of laying and bumblefoot favor keel bone fractures. Animals.

[bib0011] Harlander-Matauschek A., Rodenburg T.B., Sandilands V., Tobalske B.W., Toscano M.J. (2015). Causes of keel bone damage and their solutions in laying hens. Worlds Poult. Sci. J..

[bib0012] Henriques J.F., Caseiro R., Martins P., Batista J. (2015). High-speed tracking with kernelized correlation filters. IEEE Trans. Pattern Anal. Mach. Intell..

[bib0013] Jones R.B., Faure J.M. (1981). The effects of regular handling on fear responses in the domestic chick. Behav. Process..

[bib0014] Minville V., Laffosse J.M., Fourcade O., Girolami J.P., Tack I. (2008). Mouse model of fracture pain. Anesthesiology.

[bib0015] Nakano T., Ikawa N.I., Ozimek L. (2003). Chemical composition of chicken eggshell and shell membranes. Poult. Sci..

[bib0016] Nasr M.A.F., Browne W.J., Caplen G., Hothersall B., Murrell J.C., Nicol C.J. (2013). Positive affective state induced by opioid analgesia in laying hens with bone fractures. Appl. Anim. Behav. Sci..

[bib0017] Nasr M.A.F., Murrell J., Wilkins L.J., Nicol C.J. (2012). The effect of keel fractures on egg-production parameters, mobility and behaviour in individual laying hens. Anim. Welfare.

[bib0018] Nasr M.A.F., Nicol C.J., Murrell J.C. (2012). Do laying hens with keel bone fractures experience pain?. PLoS One.

[bib0019] Nasr M.A.F., Nicol C.J., Wilkins L., Murrell J.C. (2015). The effects of two non-steroidal anti-inflammatory drugs on the mobility of laying hens with keel bone fractures. Vet. Anaesth. Analg..

[bib0020] Nicol C.J., Caplen G., Edgar J., Browne W.J. (2009). Associations between welfare indicators and environmental choice in laying hens. Anim. Behav..

[bib0021] Parent A.J., Beaudet N., Beaudry H., Bergeron J., Bérubé P., Drolet G., Sarret P., Gendron L. (2012). Increased anxiety-like behaviors in rats experiencing chronic inflammatory pain. Behav. Brain Res..

[bib0022] Rentsch A.K., Rufener C.B., Spadavecchia C., Stratmann A., Toscano M.J. (2019). Laying hen's mobility is impaired by keel bone fractures and does not improve with paracetamol treatment. Appl. Anim. Behav. Sci..

[bib0023] Riber A.B., Casey-Trott T.M., Herskin M.S. (2018). The influence of keel bone damage on welfare of laying hens. Front Vet Sci.

[bib0024] Richards G.J., Nasr M.A., Brown S.N., Szamocki E.M.G., Murrell J., Barr F., Wilkins L.J. (2011). Use of radiography to identify keel bone fractures in laying hens and assess healing in live birds. Vet. Rec..

[bib0025] Richards G.J., Wilkins L.J., Knowles T.G., Booth F., Toscano M.J., Nicol C.J., Brown S.N. (2012). Pop hole use by hens with different keel fracture status monitored throughout the laying period. Vet. Rec..

[bib0026] Rufener C., Abreu Y., Asher L., Berezowski J.A., Sousa F.M., Stratmann A., Toscano M.J. (2019). Keel bone fractures are associated with individual mobility of laying hens in an aviary system. Appl. Anim. Behav. Sci..

[bib0027] Rufener C., Makagon M.M. (2020). Keel bone fractures in laying hens: a systematic review of prevalence across age, housing systems, and strains. J. Anim. Sci..

[bib0028] Santy J., Mackintosh C. (2001). A phenomenological study of pain following fractured shaft of femur. J. Clin. Nurs..

[bib0029] Sieberg C.B., Taras C., Gomaa A., Nickerson C., Wong C., Ward C., Baskozos G., Bennett D.L.H., Ramirez J.D., Themistocleous A.C., Rice A.S.C., Shillo P.R., Tesfaye S., Edwards R.R., Andrews N.A., Berde C., Costigan M. (2018). Neuropathic pain drives anxiety behavior in mice, results consistent with anxiety levels in diabetic neuropathy patients. Pain Rep..

[bib0030] Thiruvenkadan A.K., Panneerselvam S., Prabakaran R. (2010). Layer breeding strategies: an overview. World's Poult. Sci. J..

[bib0031] Toscano M., Booth F., Richards G., Brown S., Karcher D., Tarlton J. (2018). Modeling collisions in laying hens as a tool to identify causative factors for keel bone fractures and means to reduce their occurrence and severity. PLoS One.

[bib0032] Wei H., Feng Y., Ding S., Nian H., Yu H., Zhao Q., Bao J., Zhang R. (2022). Keel bone damage affects behavioral and physiological responses related to stress and fear in two strains of laying hens. J. Anim. Sci..

[bib0033] Wilkins L.J., Brown S.N., Zimmerman P.H., Leeb C., Nicol C.J. (2004). Investigation of palpation as a method for determining the prevalence of keel and furculum damage in laying hens. Vet. Rec..

[bib0034] Wilkins L.J., McKinstry J.L., Avery N.C., Knowles T.G., Brown S.N., Tarlton J., Nicol C.J. (2011). Influence of housing system and design on bone strength and keel bone fractures in laying hens. Vet. Rec..

